# Integrated hormonal, metabolic, and epigenetic regulation of corpus luteum function: insights into steroidogenesis, angiogenesis, and regression

**DOI:** 10.3389/fendo.2026.1812524

**Published:** 2026-04-27

**Authors:** Muhammad Tariq, Mohamed Tharwat, Shumaila Batool, Abdul Quddus, Muhammad Safdar, Yousef M. Alharbi

**Affiliations:** 1 College of Animal Science and Technology, Nanjing Agricultural University, Nanjing, Jiangsu, China; 2 Department of Clinical Sciences, College of Veterinary Medicine, Qassim University, Buraidah, Saudi Arabia; 3 College of Life Sciences and Technology, Tarim University, Alar, Xinjiang, China; 4 Department of Animal Reproduction, Lasbela University of Agriculture Water and Marine Sciences, Uthal, Balochistan, Pakistan; 5 Foundation for Research and Technology-Hellas, Institute of Molecular Biology and Biotechnology, Heraklion, Crete, Greece; 6 Department of Medical Biosciences, College of Veterinary Medicine, Qassim University, Buraidah, Saudi Arabia

**Keywords:** AMP-activated protein kinase, angiogenesis, corpus luteum, epigenetics, luteolysis, peroxisome proliferator-activated receptor gamma, progesterone, steroidogenesis

## Abstract

The corpus luteum is a transient endocrine structure essential for establishing and maintaining early pregnancy, primarily through the secretion of progesterone. Its function is regulated by a complex interplay of hormonal, metabolic, and epigenetic factors that govern steroidogenesis, angiogenesis, and luteal cell fate. Luteotrophic hormones such as luteinizing hormone, prolactin, and prostaglandin E2 promote corpus luteum maintenance by activating key signaling pathways, including cyclic adenosine monophosphate/protein kinase A and mitogen-activated protein kinase, while luteolytic factors such as prostaglandin F2α initiate regression through calcium- and protein kinase C-mediated pathways. Recent studies have highlighted the central roles of metabolic regulators AMP-activated protein kinase and Peroxisome proliferator-activated receptor gamma and the histone methyltransferase Enhancer of zeste homolog 2 in modulating corpus luteum function. These molecules integrate hormonal signals with intracellular energy and lipid status, ultimately influencing gene expression through transcriptional and epigenetic mechanisms. Epigenetic modifications, including DNA methylation, histone acetylation/methylation, and microRNAs, act as dynamic regulators of genes involved in steroid biosynthesis, angiogenesis, and programmed cell death. This review highlights the convergence of hormonal, metabolic, and epigenetic pathways in controlling corpus luteum function and regression, offering new insights into reproductive regulation and therapeutic targets for luteal insufficiency.

## 1 Introduction

The corpus luteum (CL) is a temporary endocrine organ that forms in the mammalian ovary following ovulation, during the luteal phase of the estrous or menstrual cycle. Its major task is the production and release of progesterone (P_4_), a steroid hormone essential for priming the uterine endometrium for implantation and maintaining early pregnancy (1). Progesterone promotes several processes necessary for successful gestation, including endometrial differentiation, immune tolerance toward the embryo, suppression of uterine contractions, and support for placental development (2). In most mammalian species, pregnancy termination is closely linked to luteolysis, an involutional process that is greatly regulated by the uterine-derived prostaglandin F2α (PGF2α) serving as the major luteolytic hormone to trigger both functional and structural regression of the corpus luteum. Recent studies have highlighted the role of vascular regression (angioregression) and endothelial remodeling as key mechanisms underlying corpus luteum regression (3). This process leads to decreased levels of circulating progesterone and prepares the body for the next reproductive cycle (4). However, in the event of fertilization and implantation, maternal recognition of pregnancy involves endocrine signals (e.g., chorionic gonadotropin in humans or interferon tau in ruminants), which prevent luteolysis and maintain luteal progesterone production until placental takeover occurs (5, 6). The lifespan and functionality of the CL are therefore tightly regulated, and its proper development and maintenance are essential for reproductive success across mammalian species, including humans, primates, rodents, and domestic animals.

**Figure 1:**
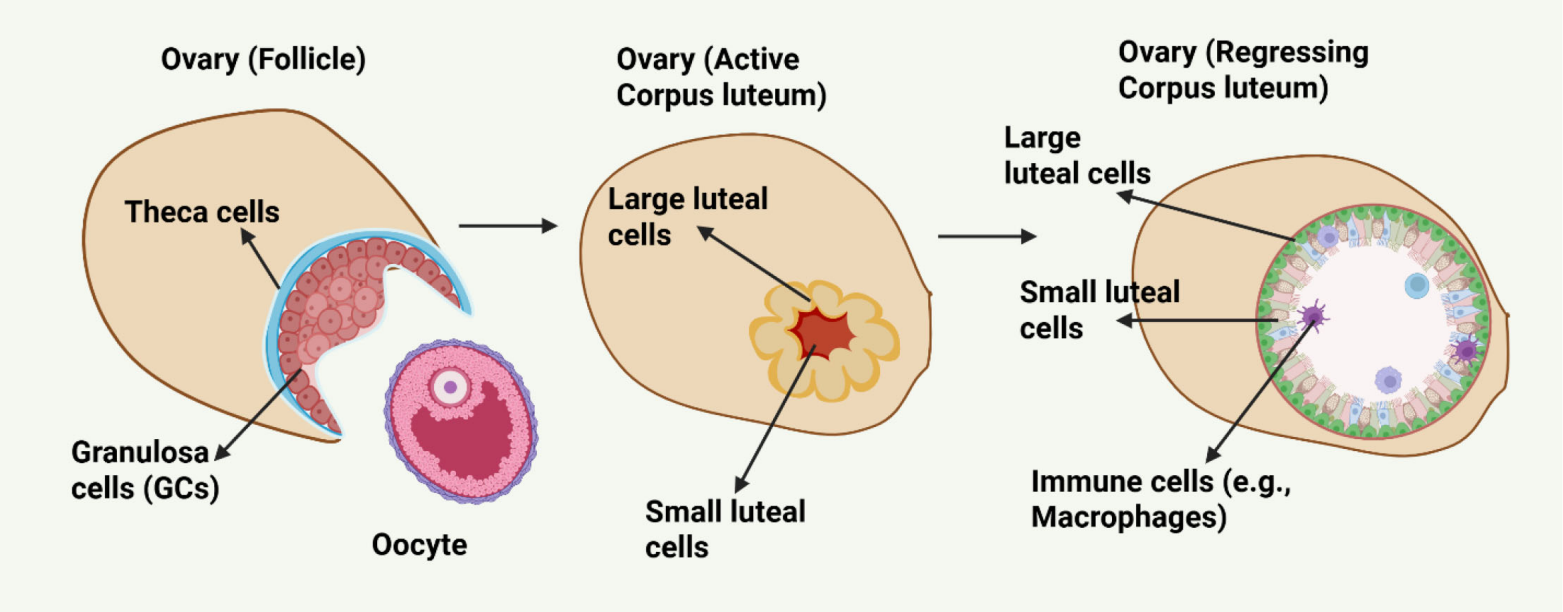
Schematic representation of follicular development and corpus luteum formation, highlighting cellular differentiation and structural changes. The image was created by the authors using BioRender.com and is conceptually based on previously published studies (7). The upper panel depicts how the CL comes into existence upon ovulation. The residual theca cells differentiate into small luteal cells and mural GCs differentiate into steroidogenic, active large luteal cells. Together these cells form the CL, a highly vascularized, steroidogenic organ. During luteal regression, luteal cells stop producing hormones. Luteal cells gradually undergo degeneration, and the CL is invaded by immune cells, including macrophages.

Genetic and epigenetic networks regulate the development, maintenance, and regression of the corpus luteum (CL). The luteinization process, which is the conversion of granulosa and theca cells of a ruptured follicle (**Figure 1****),** is initiated and maintained by the luteinizing hormone (LH) surge. This hormonal signal triggers intracellular signaling cascades through cyclic AMP (cAMP), protein kinase A (PKA) and subsequent transcription factors that act together to regulate steroidogenic gene expression (8). Progesterone synthesis is coordinated by several important regulators, such as steroidogenic acute regulatory protein (STAR), CYP11A1, and HSD3B. They increase their expression at the time of corpus luteum formation (9).

odern research suggests that metabolic regulators such as AMP-activated protein kinase (AMPK) and transcriptional modulators like peroxisome proliferator-activated receptor gamma (PPARγ) orchestrate steroidogenic gene expression through their interactions with hormonal signals (LH, PGF2α) and epigenetic modifiers such as enhancer of zeste homolog 2 (EZH2) (10, 11). These pathways collectively integrate endocrine, metabolic, and chromatin-level regulation, offering novel insights into how the CL supports progesterone production, undergoes regression, or does not support pregnancy. Understanding this integrated regulatory network is essential for unraveling the mechanisms of luteal biology and finding therapeutic targets for fertility management.

Recent empirical evidence suggests that epigenetic processes, such as DNA methylation, histone modifications, and non-coding RNAs represent important control layers in the biology of the corpus luteum (12). DNA methylation tends to inhibit the expression of genes and has the potential to regulate both the steroidogenesis of the luteal phase and apoptosis. As an illustration, active promoter methylation changes of steroidogenic genes have been associated with the functional status of the bovine and human corpus luteum (13). Post-translational modifications of histones, especially acetylation and methylation, play a key role in the control of chromatin structure and accessibility to transcription-factors. The enzyme machineries that effect these changes, histone acetyltransferases (HATs) and histone deacetylases (HDACs), are expressed in luteal tissues, and are believed to regulate gene expression during both luteal growth and luteolysis (14, 15).

Non-coding RNA elements, especially microRNAs (miRNAs) tightly regulate the principal luteal transcriptome. There are also indications that certain miRNAs that are expressed in the corpus luteum, namely miR-21, miR-34a and miR-378, regulate the pathway of angiogenesis, cell proliferation and apoptosis in the endocrine organ (16, 17). Long non-coding RNAs (lncRNAs) have also been detected in luteal tissues and may influence gene transcription, RNA splicing, and nuclear architecture, although their functions in the CL remain under investigation (18, 19). Altogether, these epigenetic mechanisms integrate hormonal cues with the transcriptional machinery to ensure the timely differentiation, function, and regression of the CL (7), thus supporting normal reproductive physiology in mammals.

Given its vital role, any dysfunction in CL development or maintenance can have serious consequences for pregnancy outcomes. CL insufficiency marked by inadequate progesterone production, is a common cause of early pregnancy loss in both humans and domestic animals (20–22). In livestock, suboptimal luteal function leads to reduced conception rates, embryonic mortality, and economic losses (23). Several factors contribute to CL dysfunction, including hormonal imbalances, poor vascularization, immune dysregulation, and metabolic stress (21, 24). Deficient expressions of steroidogenic genes or signaling molecules like VEGF (vascular endothelial growth factor) can impair angiogenesis, reducing the delivery of cholesterol and trophic factors necessary for progesterone synthesis. Chronic stress and inflammation may activate apoptotic pathways or suppress LH signaling, promoting premature luteolysis (25).

Epigenetic dysregulation has become one of the most important factors in the functioning of the corpus luteum (CL), especially in the regulation of steroidogenic gene activity, angiogenesis, and luteal cell survival. Defective production of progesterone and luteal insufficiency have been linked to aberrant DNA methylation of major steroidogenic genes including STAR, CYP11A1 and HSD3B. Also, the changes in microRNA expression may interfere with the pro-survival versus pro-apoptotic signaling pathways in the luteal cells (26, 27). Environmental exposures, endocrine-disrupting chemicals, and nutritional deficiencies may trigger such epigenetic alterations, resulting in impaired luteal function and compromised fertility (28, 29). Reproductive dysfunction is a major issue in human and agricultural applications, and an in-depth knowledge of the underlying molecular pathology is essential in the design of effective diagnostic biomarkers and specific therapeutic interventions. The corpus luteum (CL) is a critical structure for supporting pregnancy; however, the combined genetic and epigenetic mechanisms that coordinate their activities have not been well understood in mammalian species. Many of the existing studies analyze single molecular layers, and the gap in understanding how integrated networks influence the development of the luteal system, its secretion, and regression remains open. In addition, the molecular mechanism of CL dysfunction and its implication in pregnancy loss has not been given enough emphasis.

This review highlights the genetic, metabolic, and epigenetic regulation of corpus luteum (CL) function, emphasizing how luteotrophic and luteolytic signals (LH, PGF2α, adiponectin) converge on key regulators such as AMPK, PPARγ, and EZH2. Disruptions in these pathways underline luteal insufficiency and reproductive failure. By focusing on the AMPK–PPARγ–EZH2 axis, this review identifies potential molecular targets for improving fertility outcomes, while also underscoring how genomic and epigenomic insights are advancing reproductive biology and guiding novel therapeutic strategies.

## 2 Genetic regulation of corpus luteum development

### 2.1 Important genes in CL formation and maintenance

Luteinization, steroidogenesis and luteal maintenance in mammals rely on a complex system of genetic factors that control the creation and activity of the corpus luteum (7). In this network, the luteinizing hormone receptor (LHCGR), steroidogenic acute regulatory protein (STAR), and progesterone receptor (PGR) are in the central position (30). In addition to these genes, upstream signaling pathways such as AMPK–mTOR and PPARγ have been shown to regulate the transcriptional activity of steroidogenic genes like STAR and HSD3B, directly or indirectly through epigenetic enzymes such as EZH2 (**Table 1**) (41).

**Table 1:** Key genes involved in corpus luteum development and function.

Gene	Full name	Function in CL	Signaling pathway	Impact on pregnancy	Reference
LHCGR	Luteinizing Hormone Receptor	Mediates LH signaling essential for luteinization and progesterone production	cAMP/PKA, MAPK	Deficiency leads to luteal insufficiency and implantation failure	(31, 32)
STAR	Steroidogenic Acute Regulatory Protein	Transports cholesterol into mitochondria, initiating steroidogenesis	cAMP/PKA	Crucial for progesterone synthesis; defects cause early pregnancy loss	(33, 34)
PGR	Progesterone Receptor	Mediates progesterone action in luteal cells, essential for CL maintenance	Genomic signaling	Downregulation linked with early CL regression	(35)
SF-1	Steroidogenic Factor-1 (NR5A1)	Regulates expression of steroidogenic genes including STAR and CYP11A1	Nuclear receptor signaling	Control luteal function and progesterone production	(36, 37)
FOXO1	Forkhead Box O1	Controls apoptosis and proliferation in luteal cells	PI3K/AKT, FOXO signaling	Altered expression linked with CL regression	(38, 39)
NR5A2	Nuclear Receptor Subfamily 5 Group A Member 2	Activates luteinization-related genes	WNT/β-catenin	Impaired function causes defective CL formation	(40)

Luteinizing hormone receptor (LHCGR) also known as luteinizing hormone/chorionic gonadotropin receptor (LHCGR), is a G protein-coupled receptor in the superfamily and is expressed in theca and granulosa cells of the mammalian ovary. Luteinizing hormone (LH) and human chorionic gonadotropin (hCG) through LHCGR play their physiological role, which triggers cascades of signals that are essential to ovulation and corpus luteum (CL) development (42). The LH or hCG binding to the LHCGR leads to activation of the adenylate cyclase pathway, which raises the cyclic AMP (cAMP) and so activates protein kinase A (PKA). PKA subsequently phosphorylates cAMP-responsive elements (CRE) binding proteins (CREB) which results in upregulation of transcription of genes involved in steroidogenesis (43).

**Figure 2:**
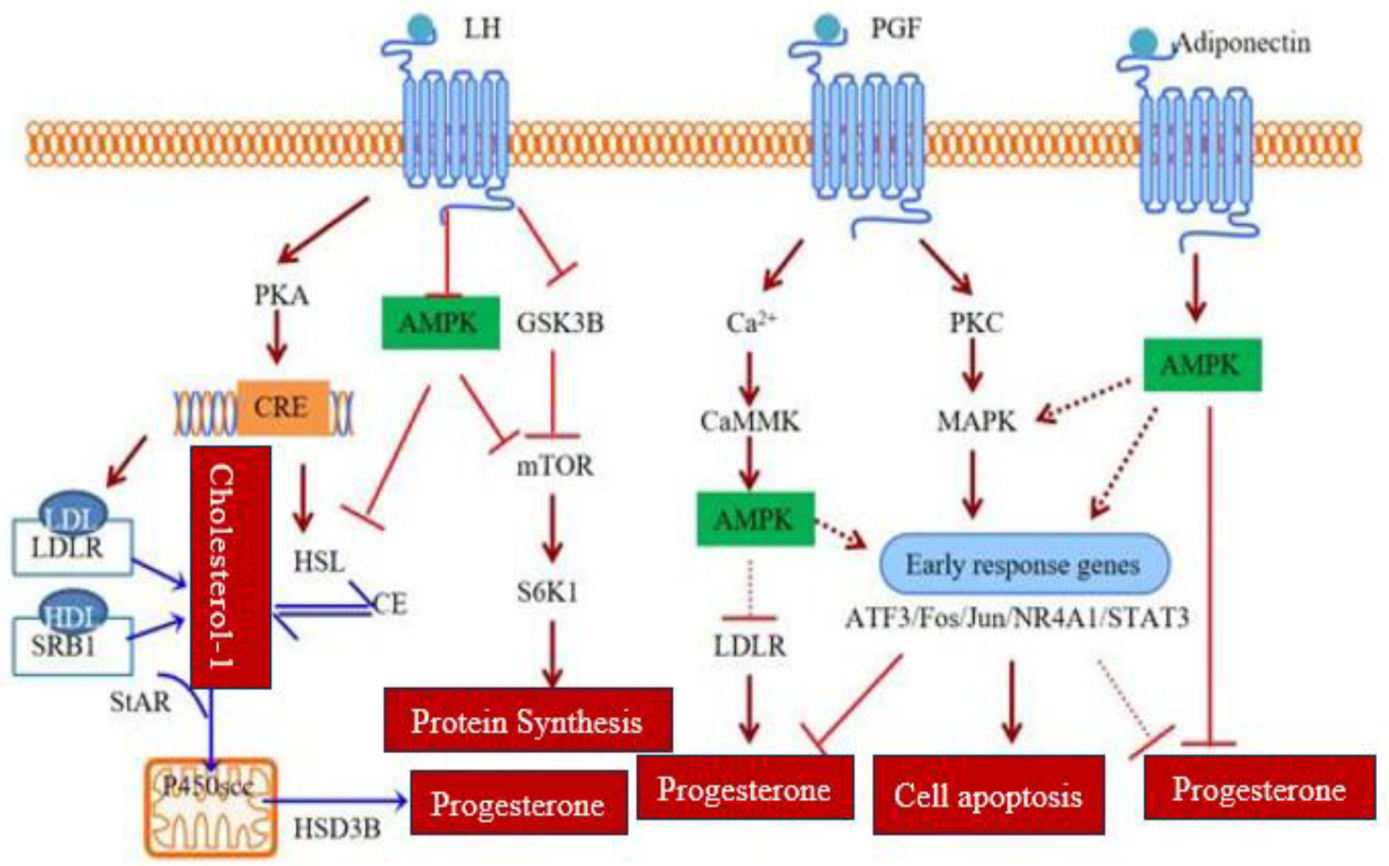
Schematic illustration of signaling pathways regulating progesterone synthesis, metabolism, and apoptosis in the corpus luteum. The image was created by the authors using BioRender.com and is conceptually based on previously published studies (35, 39). Integrated signaling pathways regulating corpus luteum function. LH, PGF_2_α, and adiponectin activate PKA-PKC/MAPK-, Ca²^+^-, and AMPK-dependent pathways to modulate cholesterol transport, protein synthesis, progesterone production, and luteal cell survival or apoptosis.

The expression of LHCGR is highly variable in a cyclic manner. The FSH and estradiol pre-ovulatory stimulate the granulosa cell LHCGR mRNA levels and ready the cells to the LH surge (44). Granulosa cells, after ovulation, become luteal cells and in differentiation continue to express LHCGR enabling the production of progesterone to be maintained. During anovulatory cycles or luteal regression after ovulation, the decreasing LH is related to a decreased LHCGR expression (45). On the other hand, hCG maintains the expression of LHCGR in early pregnancy, extending the life of the CL and the production of progesterone (46). LH signaling activates both the PKA and AMPK pathways. AMPK, in turn, inhibits GSK3B and activates mTOR signaling, promoting steroidogenesis by enhancing STAR and HSD3B expression (**Figure 2****).**

STAR plays the central role of mediating the acute control of synthesis of steroid hormones by allowing the transport of cholesterol between the outer and inner mitochondrial membranes, where cholesterol side-chain cleavage occurs by P450scc (47, 48). This is the rate limiting step of the steroidogenic pathway. In the mammalian corpus luteum, the expression of STAR is closely associated with the production of progesterone: during the early and mid-luteal phases, the concentrations of STAR mRNA and protein increase, and during the late luteal phase, the concentrations decrease, which reflects the reduced steroidogenic activity.

Progesterone receptor (PGR) is a hormone that mediates the effects of progesterone that is essential in the establishment and maintenance of a mammalian pregnancy (49). The receptor exists in several isoforms with the most characterized being PGR-A and PGR-B. Both isoforms act as ligand-activated transcription factors which control gene expression in different ways (50). PGR-B is mainly a transcriptional activator and PGR-A can suppress the activity of PGR-B. Their ratio varies during the estrous cycle and pregnancy and alters cellular responses to progesterone (51). PGR expression is upregulated in the corpus luteum by the LH surge and is critical to ovulation, luteinization and sustaining luteal function.

### 2.2 Role of transcription factors

A complex network of transcription factors coordinate the development and maintenance of the corpus luteum (CL) in mammals, by controlling the expression of genes necessary in the formation of the CL, steroid production and regression of the CL. Steroidogenic Factor-1 (SF-1, encoded by NR5A1), Forkhead box O1 (FOXO1) and Liver receptor homolog-1 (LRH-1, encoded by NR5A2) play essential functional roles within this network (52, 53).

SF-1 is a key nuclear receptor transcription factor, which controls the development and the functionality of steroidogenic tissues including the adrenal glands, and gonads and is coded by the NR5A1 gene (54). In the ovary, SF-1 is essential to the generation and preservation of the CL, a transient endocrine organ that produces progesterone and causes pregnancy to be established (55). The regulatory action of SF-1 is through binding to specific DNA response factors that regulate the transcription of genes that are essential in steroidogenesis and some of these genes include CYP11A1 and Steroidogenic Acute Regulatory protein (STAR) (37). CYP11A1 converts cholesterol to pregnenolone, which is the initial stage of steroid hormone synthesis (33), whereas STAR mediates the transfer of cholesterol into mitochondria, which is a requirement of steroidogenesis (56). The ability of SF-1 to activate these genes thus ensures that the CL has the ability of producing sufficient levels of progesterone required to sustain early pregnancy.

Steroid-genic factor-1 (SF-1) transcription factor is upregulated in the process of granulosa-cell differentiation and luteinization, which in turn suggests that SF-1 is essential to the process of specification of granulosa-cell into luteal cells (57). The result of the conditional knockout experiment of SF-1 in mice granulosa cells indicates that the loss of this factor in granulosa cells triggers impaired cumulus expansion, ovulatory impairment, and reduced corpus luteum formation, which altogether suppresses progesterone production (58, 59). Such data highlights the important role of SF-1 in the formation of corpus luteum and its activity.

**Figure 3:**
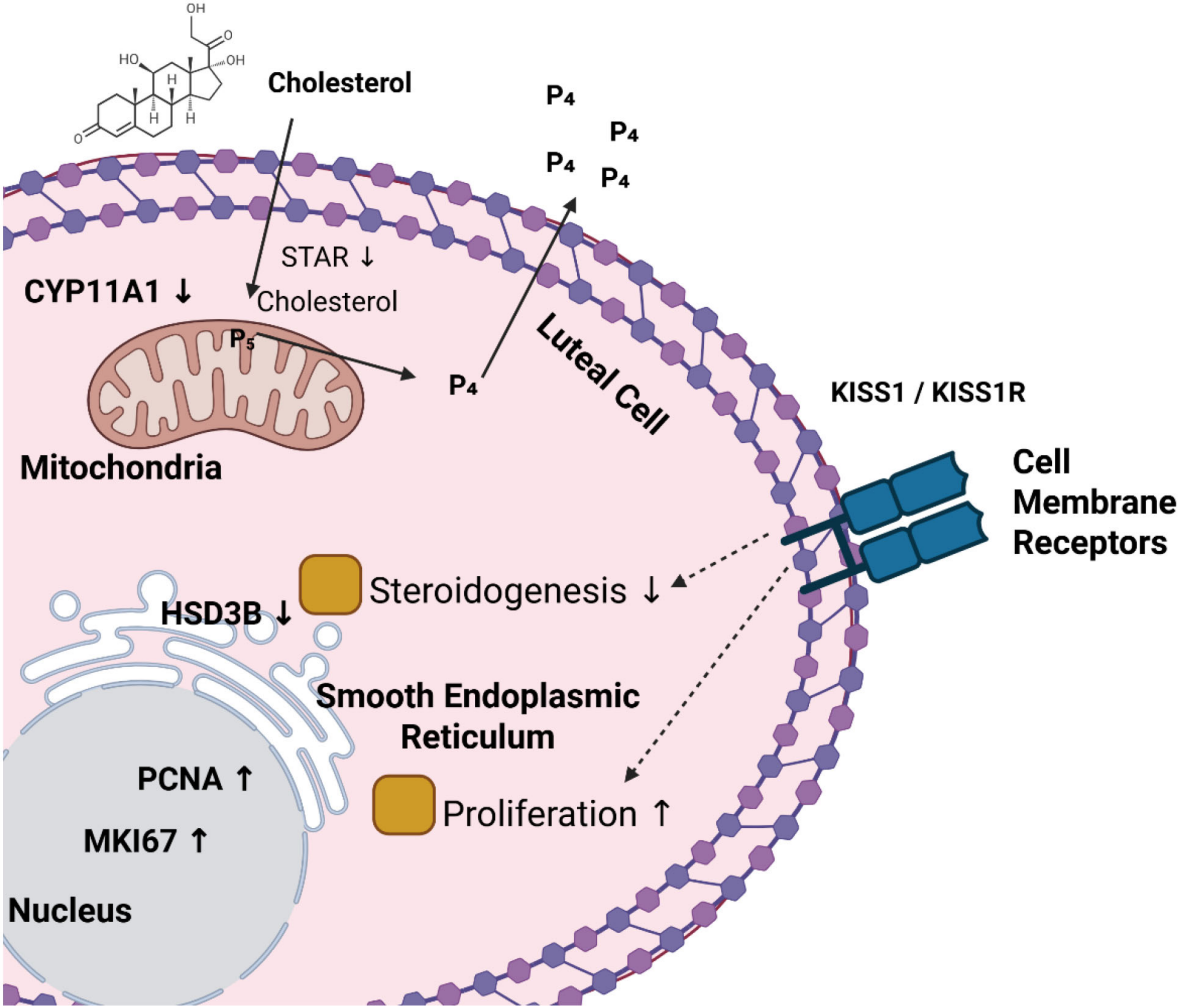
Schematic illustration of cholesterol transport, steroidogenesis, and proliferative signaling in luteal cells. The image was created by the authors using BioRender.com and is conceptually based on previously published studies (60, 61). KISS1/KISS1R Luteal cell signaling. Kisspeptin (KISS1) interacts with KISS1R and triggers Gq/11-PLC signaling to produce IP3 and DAG to stimulate Ca_2_ presence and activation of PKC. These stimulate downstream MAPK/ERK and PI3K/AKT signaling, which eases expression of luteal steroidogenic genes, cell survival and progesterone.

SF-1 regulation is performed in two ways: direct transcriptional activity and associations with various co-regulators and signaling cascades. As an example, cAMP/PKA pathway, which is stimulated in response to luteinizing hormone (LH), increases SF-1 transcriptional activity, which, in turn, increases expression of steroidogenic genes (**Figure 3**) (62). SF-1 stability, intracellular localization and protein-protein interactions are regulated by post-translational modifications, in particular phosphorylation and acetylation, which fine-tune its regulatory potential (63). The importance of SF-1 to the ovarian function can also be illustrated by human mutations in the NR5A1 gene: the mutations cause disorders of premature ovarian insufficiency (POI) thus connecting the dysregulation of SF-1 with low ovarian reserve and infertility (64, 65). These results make SF-1 a central determinant of ovarian maintenance and a potential target of fertility-related treatments.

### 2.3 Molecular pathways regulating CL function

#### 2.3.1 MAPK/ERK signaling

**Figure 4:**
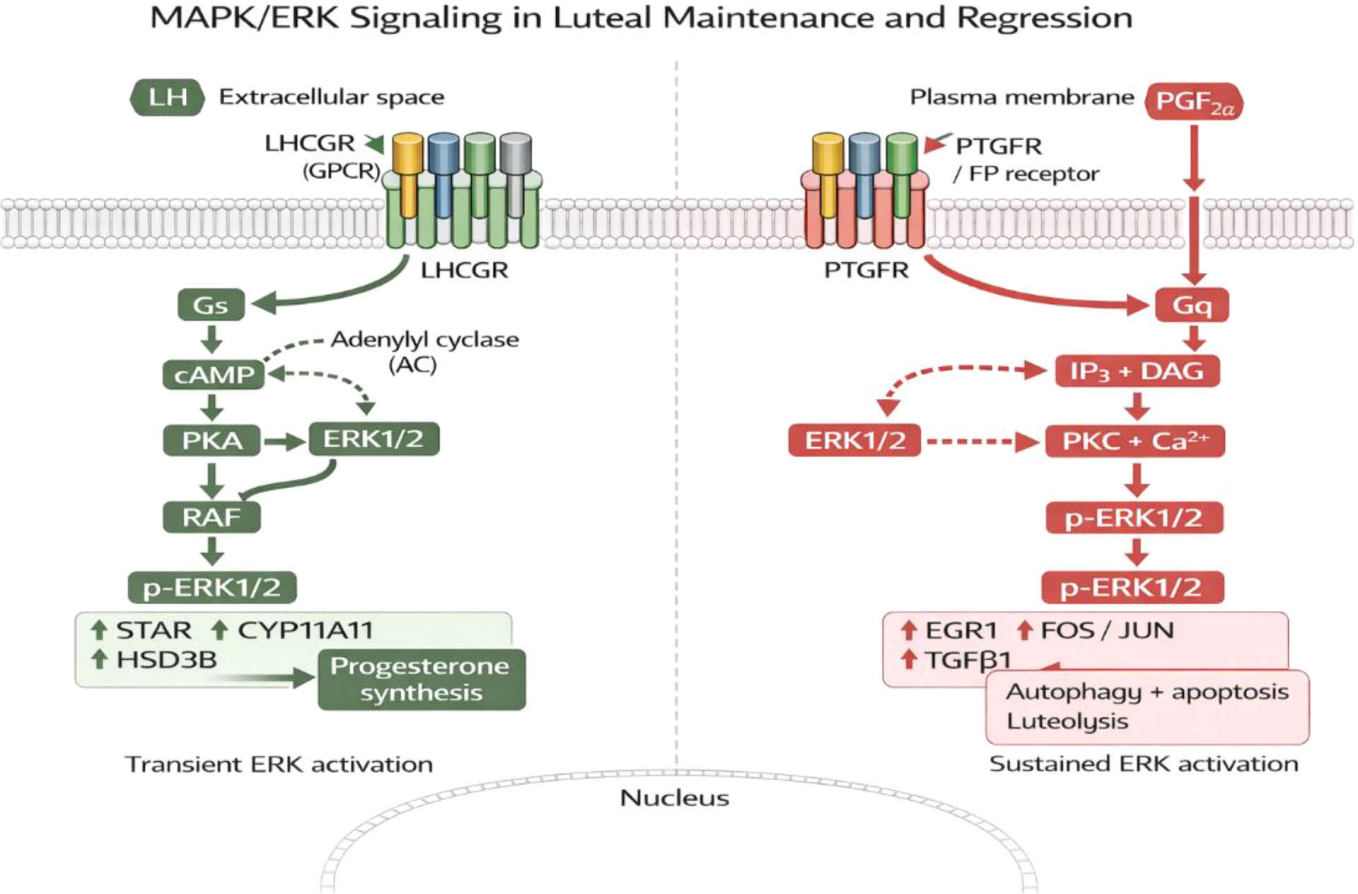
Schematic representation of MAPK/ERK signaling pathways in luteal maintenance and regression. The image was created by the authors using BioRender.com and is conceptually based on previously published studies (39, 60). MAPK/ERK luteal maintenance and luteal regression. LH stimulates LHCGR to increase the stimulation of steroidogenic gene expression (STAR, CYP11A1, HSD3B) and production of progesterone via cAMP/PKA-dependent ERK1/2 signaling through luteal maintenance. Conversely, PGF2α/activate PTGFR/FP receptor and Gq-dependent pathway to mediate ERK1/2 persistent phosphorylation to increase luteolytic mediators (EGR1, FOS/JUN, TGFb1) and autophagy/apoptosis resulting in luteolysis.

The mitogen-activated protein kinase/extracellular signal-regulated kinase (MAPK/ERK) mediates a luteotrophic and luteolytic signaling in the corpus luteum (CL) (66). LHCGR also activates MAPK/ERK through luteinizing hormone (LH) signaling to increase steroidogenic gene expression (STAR and CYP11A1) and promote the production of progesterone during the functional luteal phase. On the other hand, MAPK/ERK signaling is also recruited by prostaglandin F2 (PGF2α) which instead shifts its action to luteal destruction by activating transcription factors like EGR1 and pro-apoptotic mediators (66). MAPK/ERK thus demonstrates the effects which are context-dependent, either maintaining or regressing the luteal depending on the prevailing endocrine environment (**Figure 4**).

#### 2.3.2 PI3K/AKT signaling

**Figure 5:**
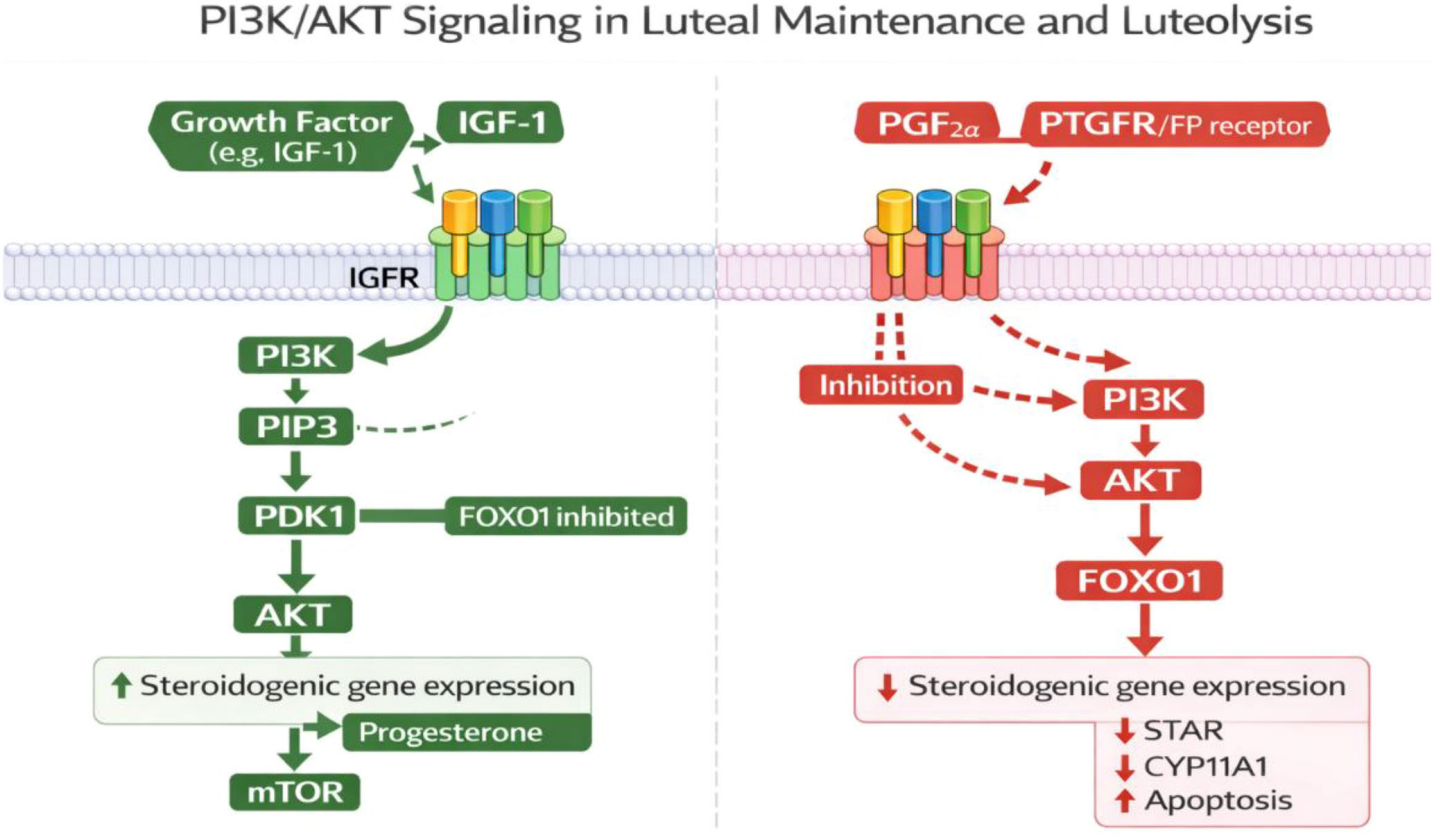
Schematic representation of PI3K/AKT signaling pathways in luteal maintenance and luteolysis. The image was created by the authors using BioRender.com and is conceptually based on previously published studies (69, 70). Luteal maintenance and luteolysis PI3K/AKT signaling. IGF-1 stimulates IGFR-PI3K signaling, which causes AKT activation via PDK1. The AKT prevents FOXO1 and activates mTOR signaling to enhance steroidogenic gene expression and progesterone synthesis in luteal maintenance. PGF2α signaling through PTGFR, on the other hand, inhibits PI3K/AKT, allowing FOXO1 to act, lowering STAR and CYP11A1, and promoting apoptosis, highlighting the role of PGF2α in luteolysis.

The luteal cell survival and steroidogenesis is mainly supported by the phosphoinositide 3-kinase/protein kinase B (PI3K/AKT) pathway. PI3K/AKT signaling is stimulated by growth factors like IGF-1 and prevents the pro-apoptotic transcription factors, including FOXO1, thereby lifting the suppression of steroidogenic genes and increasing progesterone production (67). The AKT signaling also interacts with mTOR signaling pathways to sustain protein synthesis and viability of luteal cells. PGF2α inhibits PI3K/AKT in luteolysis which leads to decreased progesterone production and enhanced apoptotic signals. Therefore PI3K/AKT is a survival axis which maintains CL integrity in luteotrophic circumstances (68) (**Figure 5****).**

#### 2.3.3 WNT/β-catenin signaling

**Figure 6:**
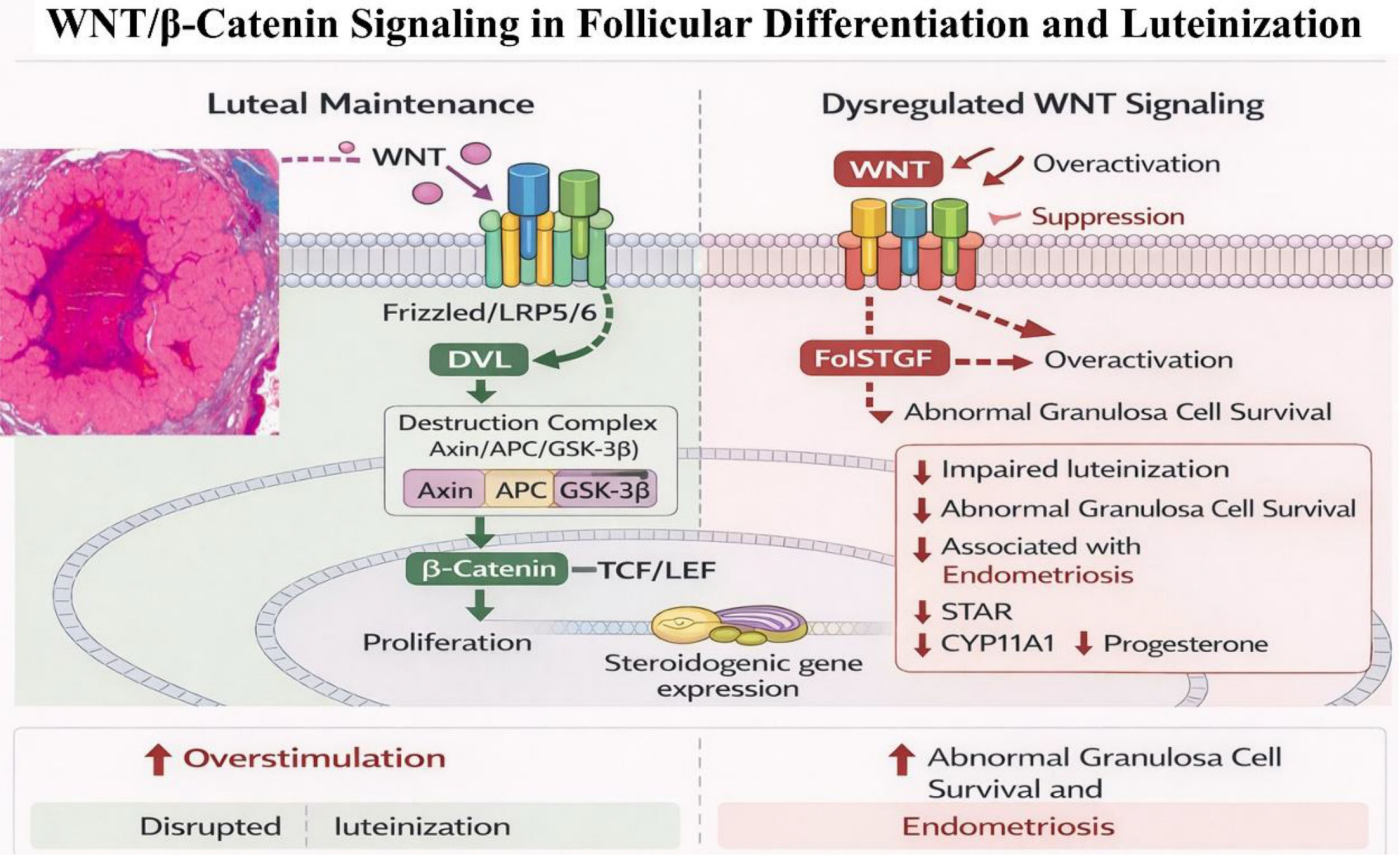
Schematic representation of WNT/β-catenin signaling in follicular differentiation and luteinization. The image was created by the authors using BioRender.com and is conceptually based on previously published studies (73, 74). WNT/β-catenin signaling during luteinization and initial development of the corpus luteum. WNT ligands bind to the receptors Frizzled (FZD) and LRP5/6, triggering Dishevelled (DVL) and suppressing the β-catenin destruction complex (Axin/APC/GSK-3β) and causing β-catenin to become stabilized. Stabilized β-catenin moves to the nucleus and binds to TCF/LEF to control the transcription of genes that govern granulosa cell proliferation, differentiation and early steroidogenic activity. Luteinization, STAR/CYP11A1 expression, and progesterone production can be dysregulated by WNT/β-catenin signaling.

WNT/β-catenin signaling pathway plays a role in follicular differentiation and luteinization transition. Activation of WNT/β-catenin in granulosa cells contributes to proliferation and expression of steroidogenic genes in early luteal formation. But this pathway should be tightly controlled because overstimulation might disrupt the process of luteinization and ovulatory (71). WNT signaling dysregulation has been linked to abnormal granulosa cell survival and reproductive pathological diseases like endometriosis (72). WNT/β-catenin signaling has a role in initial CL development that needs to be carefully regulated to enable normal luteal development (**Figure 6****).** Although these signaling pathways play essential roles in regulating corpus luteum function, their activity is further modulated by epigenetic mechanisms, which provide an additional layer of control over gene expression.

### 2.4 Angiogenesis in the CL

The process of angiogenesis is a primary and highly controlled activity in the corpus luteum (CL), which forms the basis of its rapid growth, endocrine activity, and subsequent remodeling. The CL is a highly vascularized transient endocrine structure, which requires extensive and well-orchestrated vascular remodeling following ovulation. It is triggered by the proliferation, migration, and differentiation of endothelial cells which is a complex interplay between angiogenic growth factor, hormonal signals and local microenvironmental cues. Proper vascularization is needed to provide the body with cholesterol substrates, oxygen and gonadotropins, all of which are vital to the maintenance of progesterone biosynthesis and luteal cell survival (75).

Following ovulation, the ruptured follicle undergoes rapid vascular remodeling, which is intensive endothelial cells proliferation and migration. This initial angiogenic burst is largely mediated by the vascular endothelial growth factor (VEGF) that is greatly produced on granulosa-lutein cells and theca-lutein cells due to luteinizing hormone (LH) and hypoxia-inducible factors (HIF-1α). VEGF favors endothelial cell survival, vascular permeability and capillary sprouting and fibroblast growth factor (FGF2) further stimulates endothelial proliferation and differentiation (76). This synchronized signaling leads to the development of a fine and well-permeable capillary structure, which allows the luteal functionality to be developed rapidly.

Angiogenesis is at its highest peak during the mid-luteal phase and shifts into a stabilization and maturation phase of the vessels. It is the stage of the maximal production of progesterone and complete functioning of luteal tissue. Tie2 receptor Angiopoietin-1 (ANGPT1) is a vital component in ensuring vascular integrity, pericyte recruitment and endothelial cell survival. Moreover, further endothelial maintenance, mediated by continued VEGF signaling, and additional growth factor interactions (insulin-like growth factor-1 (IGF-1) and platelet-derived growth factor (PDGF)) all promote vascular remodelling and luteal maintenance (77). The development of an unchanging microvascular framework is paramount to perpetuating the steroidogenic activity as well as luteal cell maintenance.

When there is no pregnancy, the CL experiences functional and structural regression which is closely linked with vascular destabilization. The main luteolytic factor that triggers this process is prostaglandin F2α (PGF2α) that down-regulates VEGF and up-regulates the expression of angiopoietin-2 (ANGPT2) that suppresses ANGPT1-Tie2 signaling. This change triggers the endothelial cell apoptosis, vessel regression, and blood flow reduction in the CL (78). Simultaneously, inflammatory mediators and oxidative stress also play a role in the vascular destruction and structural luteolysis. The failure of vascular support eventually results in decreased production of progesterone and the involution of the luteal tissue.

## 3 Epigenetic regulation of corpus luteum function

In addition to genetic and signaling pathways, epigenetic mechanisms act as key integrators of hormonal and metabolic signals, ensuring coordinated regulation of corpus luteum function. Epigenetic mechanisms are critical in regulating gene expression in the corpus luteum (CL), particularly in response to dynamic hormonal and metabolic changes throughout the luteal phase. Unlike genetic alterations, epigenetic modifications are reversible and responsive to physiological stimuli, making them vital regulators of luteal formation, function, and regression.

### 3.1 DNA methylation and gene expression in CL

In corpus luteum (CL) formation and functioning, epigenetic changes, and particularly DNA methylation are critical to gene expression regulation (79). DNA methylation is a key epigenetic mechanism of gene silencing in which DNA methyltransferase (DNMTs) attach a methyl group to the fifth carbon of cytosines residues in the cytosine-paired-with-guanine (CpG) dinucleotides. Three mammalian DNMTs have been defined: DNA methyltransferase 1 (DNMT1), DNA methyltransferase 3 alpha (DNMT3A), and DNA methyltransferase 3 beta (DNMT3B). DNMT1 is mainly involved in the maintenance of the pre-existing pattern of DNA methylation during replication, and DNMT3A and DNMT3B are the *de novo* methyltransferase (80). DNA methylation has also been implicated in luteal function as it has been demonstrated that methylation of the aromatase gene promoter in bovine CL represses aromatase expression (81).

Methylation of DNA normally occurs on CpG dinucleotides and is linked to transcriptional silencing (82). This epigenetic process in the bovine CL regulates important genes of steroidogenesis and angiogenesis. It has been shown that LH surge induces rapid methylation of steroidogenic acute regulatory protein (STAR), cytochrome P450 side-chain cleavage enzyme (CYP11A1), and 3beta-hydroxysteroid dehydrogenase (HSD3B) that are critical in progesterone production (83).

The epigenetic control is also found in genes that are involved in the development of the vascular system such as vascular endothelial growth factor (VEGF), among others (84). The post-ovulatory CL is characterized by rapid neovascularization which is VEGF-dependent. The methylation of the promoter region has been linked to the expression levels of the VEGF in the luteal endothelial cells. Defects in the luteal phase and infertility are associated with abnormalities in the methylation of these genes and underline the value of a strictly regulated epigenetic programming (85). The alteration of DNA methylation patterns in CL is linked to such pathological states as polycystic ovary syndrome (PCOS) and endometriosis, where the imbalance of steroidogenic genes expression results in the hormonal imbalance. On this basis, epigenetic therapy which entails DNA methylation is emerging as a potential therapy in restoration of normal CL activity in such disorders (86). In conclusion, DNA methylation is dynamic and reversible and is a component of normal CL functionality and successful maintenance of pregnancy (87).

**Figure 7:**
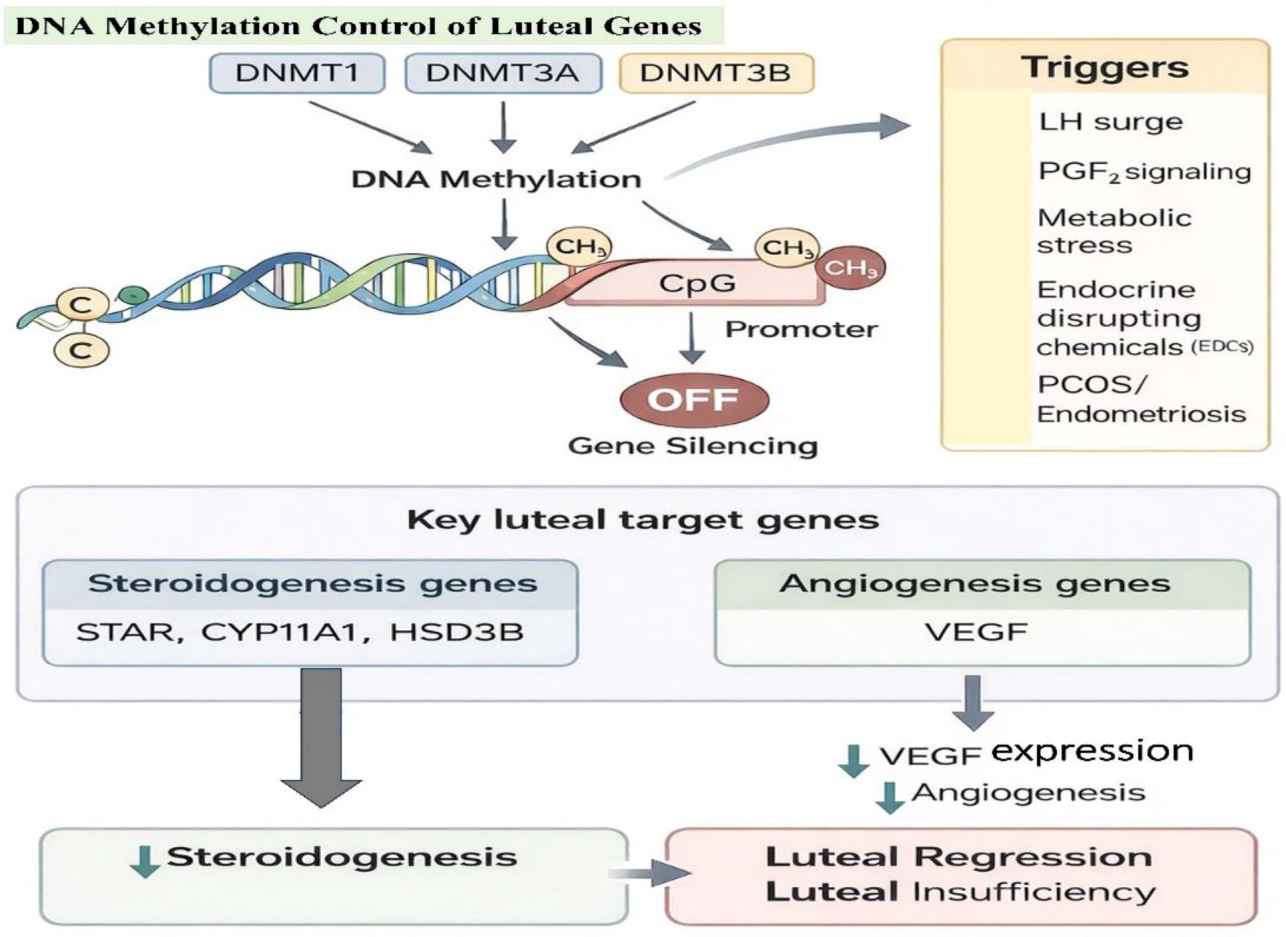
Schematic representation of DNA methylation–mediated regulation of luteal gene expression and its impact on steroidogenesis, angiogenesis, and luteal function. The image was created by the authors using BioRender.com and is conceptually based on previously published studies (7, 89). Luteal gene expression regulation by DNA methylation. CpG promoter methylation is mediated by DNMT1, DNMT3A, and DNMT3B, which result in transcriptional silencing of luteal genes. The regulation of steroidogenic genes (STAR, CYP11A1, HSD3B) and VEGF regulated by methylation affect the production of progesterone and the process of luteal angiogenesis. Methylation patterns may change in response to hormonal (LH and PGF2α), metabolic (stress), endocrine (disruptors) and reproductive (e.g., PCOS and endometriosis) causes.

In luteal cells, DNA methylation dynamically regulates the transcription of steroidogenic genes. For example, increased methylation of the PGR gene promoter has been associated with reduced progesterone receptor expression during luteal regression. Similarly, methylation of STAR or HSD3B gene promoters may restrict steroidogenesis as the CL transitions to regression (88). The methylation status of these genes can be influenced by hormonal cues such as PGF2α or metabolic stress. Studies suggest that exposure to endocrine-disrupting chemicals (EDCs) or alterations in the energy balance may also affect DNA methylation in the CL, potentially leading to luteal insufficiency (**Figure 7**) (29).

### 3.2 Histone modifications in luteal cells

Histone modifications including, acetylation and methylation, are important regulators of chromatin structure and gene accessibility in the progesterone-producing corpus luteum (CL). Such post-translational modifications of histone tails control the transcription of luteal cells and influence the functional life of the CL (90, 91). Histone acetylation by histone acetyltransferases (HATs) is likely to activate transcription by relaxing chromatin and histone deacetylation by histone deacetylases (HDACs) is likely to inhibit transcription by compacting chromatin (90). During the development of CL, higher acetylation of histone H3 at STAR, HSD3B, and CYP19A1 promoters is linked to higher expression of these steroidogenic genes (92). Conversely, CL regression is linked to increased HDAC activity and decreased acetylation which prevents the production of progesterone. Histone methylation also exhibits decisive role: in healthy luteal cells, histone H3 trimethylation at lysine 4 (H3K4me3) is associated with active transcription and histone H3 tri-methylation at lysine 27 (H3K27me3) is associated with gene repression in luteolysis (93). They have proposed that epigenetic enzymes such as EZH2 (Enhancer of Zeste Homolog 2) which is a methyltransferase that catalyzes H3K27me3 may be involved in the repression of genes during luteal regression (94, 95). Therefore, the processes of histone acetylation and methylation are dynamic events that regulate significant transitions in the development, maintenance, and regression of CL.

A key repressive mark, H3K27me3, is catalyzed by EZH2, a core component of the polycomb repressive complex 2 (PRC2). During luteal regression, EZH2 is upregulated in response to PGF2α and possibly via AMPK signaling. This leads to H3K27me3 enrichment at the promoters of HSD3B, STAR, and CYP11A1, thereby silencing their expression and reducing progesterone synthesis. Conversely, in the early luteal phase, histone acetylation at these same loci promotes transcription under the influence of LH and the cAMP/PKA signaling pathway (41). This highlights the role of histone modifications as dynamic switches regulating luteal steroidogenesis in response to the endocrine environment.

### 3.3 Non-coding RNAs role

The primary regulators of post-transcriptional gene expression in the corpus luteum (CL) are non-coding RNAs (ncRNAs), i.e., microRNAs (miRNAs) and long non-coding RNAs (lncRNAs) (96). Although they are not protein coding, these molecules control mRNA stability and translation and, therefore, control many physiological processes, including CL physiology (**Table 2**).

**Table 2:** Epigenetic modifications affecting corpus luteum function.

Epigenetic mechanism	Target/example	Effect on CL function	Associated outcome	Reference
DNA Methylation	CYP19A1, HSD3B2	Suppresses expression of steroidogenic genes	Impairs progesterone synthesis	(97)
Histone Acetylation	H3K9ac, H3K27ac	Enhances transcription of luteal maintenance genes	Prolongs CL lifespan	(14, 53)
Histone Methylation	H3K27me3, EZH2 activity	Represses steroidogenic genes	Promotes luteal regression	(14, 98)
miRNAs	miR-378, miR-21	Regulate angiogenesis and cell survival in CL	Altered miRNA expression can lead to luteal insufficiency	(99)
lncRNAs	NEAT1, MALAT1	Influence PGR and VEGF expression in luteal cells	Associated with defective CL vascularization	(100)

MiRNAs are short (~22 nucleotides) RNA molecules that can form a complex with the complementary sequences of the target mRNA in the 3 untranslated region (3 UTR) that can lead to either degradation or inhibition of translation (101). Multiple miRNAs that are identified in the CL control the genes related to steroidogenesis, cell survival, and apoptosis (102, 103). As an example, miR-378 and miR-132 have been shown to increase the survival of luteal cells and progesterone synthesis by targeting pro-apoptotic genes and increasing the expression of STAR, respectively (104).

On the other hand, miRNAs like miR-34a and miR-21, have pro-luteolytic effects. MiR-34a is an apoptotic regulator in luteal cells through repression of anti-apoptotic BCL2 mRNA, but miR-21 has mixed effects on luteal regression, promoting luteal regression in some luteal phases and species and inhibiting luteal regression in others (105). The changes in the miRNA expression patterns are linked to infertility and the adverse pregnancy outcomes, which highlights the significance of the molecules in the CL homeostasis (106).

Long non-coding RNAs (lncRNAs) are typically longer than 200 nucleotides and they control gene expression by remodeling chromatin, interference of transcription, and miRNA sponges (107). However, the role of lncRNAs in the corpus luteum remains incompletely understood and requires further investigation. Some lncRNAs such as H19 and NEAT1 have been suggested to participate in progesterone biosynthesis in luteal cells. H19 regulates the expression of IGF1R and thus the angiogenesis and steroidogenesis of the luteal (108). Epigenetic regulation by lncRNA is associated with the recruitment of chromatin modifiers to steroidogenic gene loci which influences their transcriptional output. The NEAT1 is related to nuclear paraspeckle assembly and has been implicated in the survival and activity of luteal cells (109). The aberrant expression of these lncRNAs is associated with the maldevelopment of CL and subfertility (110). Therefore, non-coding RNAs are fine tuners of gene networks that are critical in preserving CL integrity and sustaining early pregnancy.

## 4 Factors influencing genetic and epigenetic regulation of CL

### 4.1 Endocrine regulation

The corpus luteum functions as a short-lived essential endocrine gland during female reproduction because it produces progesterone to sustain early pregnancy. Endocrine regulation serves as the central mechanism to control complex genetic and epigenetic operations that guide the formation along with operational standards of the corpus luteum (7). The two essential hormonal controllers for CL development are luteinizing hormone (LH) and follicle-stimulating hormone (FSH) (111).

LH activates protein kinase A (PKA) signaling, which promotes hormone-sensitive lipase (HSL) activity by enhancing phosphorylation at Ser563 and reducing phosphorylation at Ser565. Active HSL mobilizes cholesterol from lipid droplets to support progesterone biosynthesis. Concurrently, LH suppresses AMP-activated protein kinase (AMPK) activity by inducing Ser485 phosphorylation and preventing Thr172 activation by upstream kinases (CAMKKβ, LKB1) or metabolic stress. During luteolysis, AMPK is activated via Thr172 phosphorylation, leading to inhibitory phosphorylation of HSL at Ser565, suppression of cholesterol hydrolysis, and reduced steroidogenic capacity of luteal cells.

**Figure 8:**
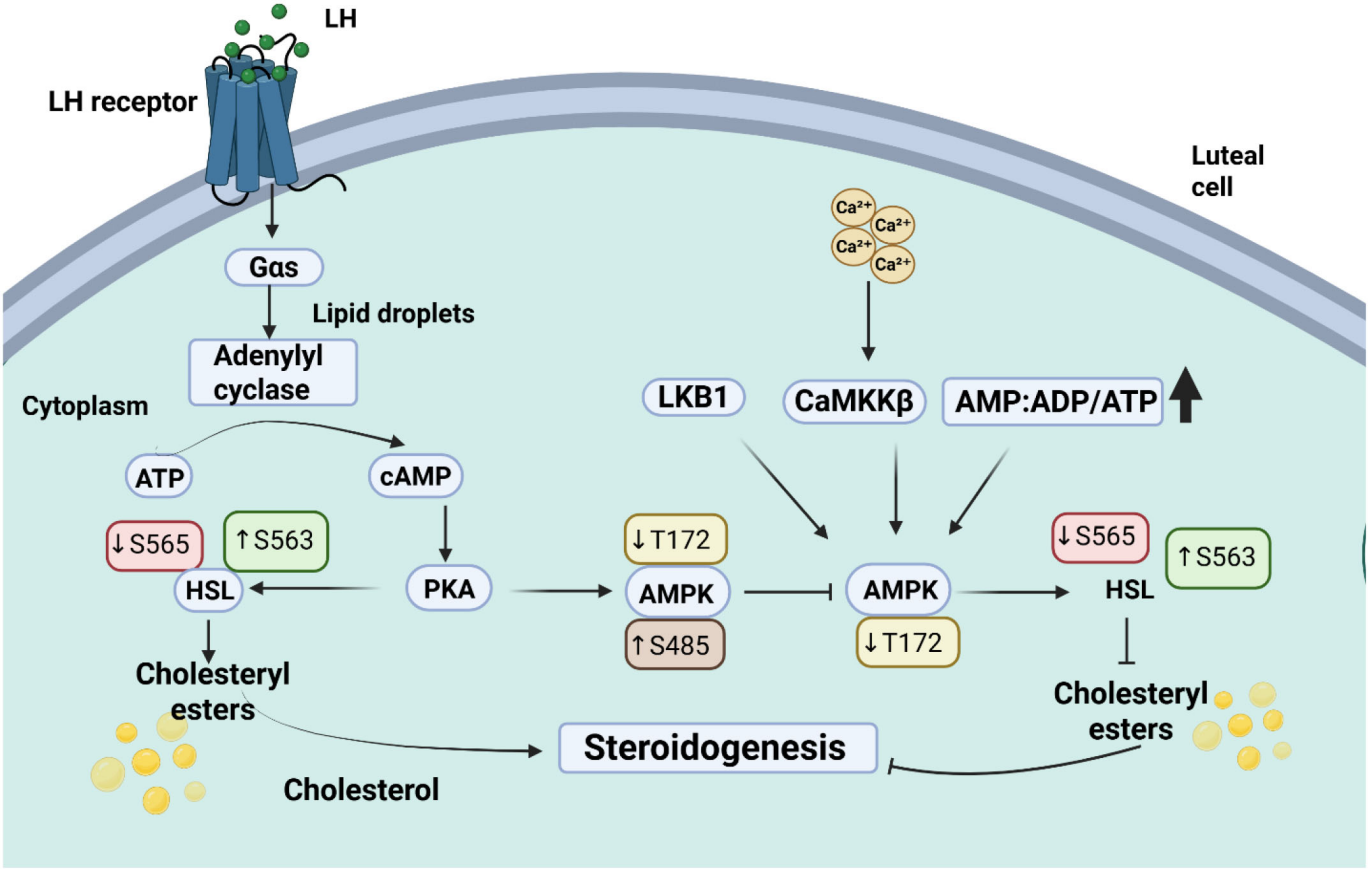
Schematic representation of AMPK and PKA signaling pathways regulating lipid metabolism and steroidogenesis in luteal cells. The image was created by the authors using BioRender.com and is conceptually based on previously published studies (112, 113). Cross talking between LH, -cAMP/PKA and AMPK signaling in luteal steroidogenesis. LH triggers the LH receptor Ga +sa enylyl cyclase pathway to elevate cAMP and activate PKA to phosphorylate and mobilize cholesterol in cholesteryl esters to generate steroids. Concurrently, cellular energy stress (AMP: ADP: ATP) and Ca²^+^ triggers AMPK through signal transduction via Thr172 phosphorylation by Ca²^+^-CaMKK 2/LKB1. HSL activity and cholesterol availability are regulated by the interaction of PKA with AMPK, thus regulating luteal steroidogenesis.

The LH surge activates ovulation while initiating the process of granulosa cell luteinization after estrogen reaches its maximum point during the late follicular phase 9 (**Figure 8**) (114). The luteinizing hormone/choriogonadotropin receptor (LHCGR) expression increases following transformation which enables downstream cyclic AMP (cAMP)/protein kinase A (PKA) pathway signaling (115). Activation of this pathway results in transcription of two vital steroidogenic genes STAR (steroidogenic acute regulatory protein) and CYP11A1 which are essential for progesterone biosynthesis in luteal cells. Genetic transcription of these imperative genes supports sufficient hormonal endometrium maintenance to ensure embryo implantation and the preservation of early pregnancy (42).

The follicular environment needs FSH to prepare it for luteinization even though FSH primarily drives folliculogenesis (116, 117). The FSH hormone controls the production of FSH receptor (FSHR) while also activating the aromatase enzyme for estrogen synthesis. The presence of estrogen affects the expression of LH receptors and prepares granulosa cells for their transformation into luteinized cells. The signaling pathway of FSH leads to consequences that affect both CL development and operation (**Table 3**) (123).

**Table 3:** Hormonal regulation of corpus luteum development.

Hormone	Role in CL development	Mechanism of action	Impact on pregnancy	References
Luteinizing Hormone (LH)	Initiates ovulation and luteinization of granulosa cells	Activates LHCGR, triggering cAMP/PKA pathway, leading to STAR and CYP11A1 expression	Essential for progesterone production, supporting early pregnancy	(39, 118)
Follicle-Stimulating Hormone (FSH)	Prepares follicle for luteinization	Stimulates FSHR expression and aromatase activity, increasing estrogen synthesis	Facilitates estrogen-driven CL development	(119, 120)
Progesterone	Maintains pregnancy by preparing endometrium for implantation	Binds to PGR, regulating immune tolerance and vascularization within CL	Crucial for embryo implantation and pregnancy maintenance	(120–122)

**Figure 9:**
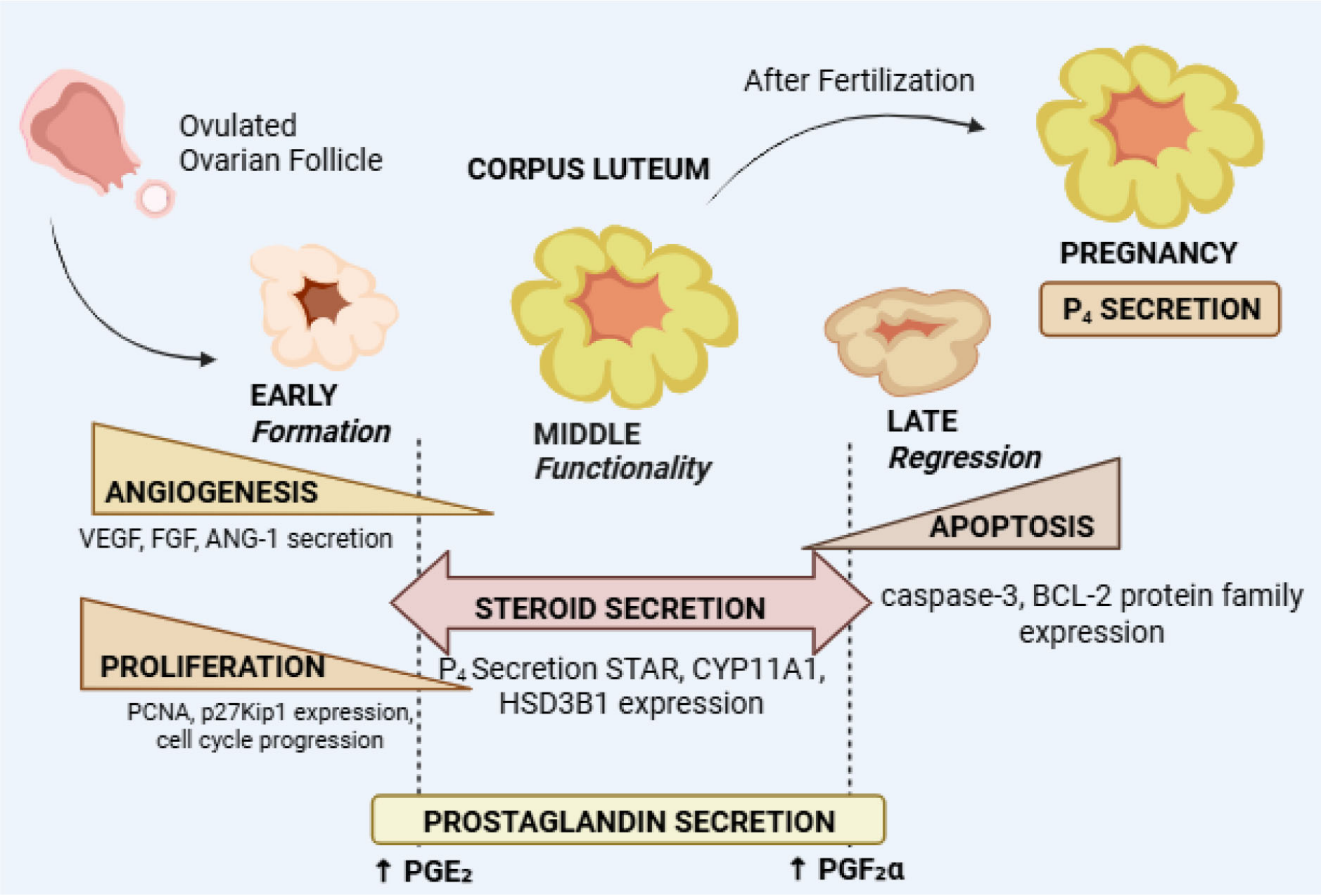
Schematic representation of corpus luteum development, function, and regression, highlighting key processes including angiogenesis, proliferation, steroidogenesis, and luteolysis. Following ovulation, the corpus luteum undergoes early development characterized by luteal cell proliferation and angiogenesis (VEGF, FGF, ANG-1). The mid-luteal phase is marked by maximal steroidogenic activity and progesterone (P4) secretion, supported by increased expression of STAR, CYP11A1, and HSD3B1. In the absence of pregnancy, the corpus luteum undergoes regression, characterized by apoptotic signaling (caspase-3, BCL-2 family) and increased PGF2α, whereas PGE2 supports luteal maintenance. Following fertilization, sustained corpus luteum activity maintains progesterone secretion necessary for pregnancy. The image was created by the authors using BioRender.com and is conceptually based on previously published studies (34, 38). The image was created by the authors using BioRender.com and is conceptually based on previously published studies (35, 129).

The epigenetic processes involving DNA methylation as well as histone modifications act as another control mechanism to handle gene accessibility and transcription (124). The sensitivity of the CL to hormonal cues decreases when LHCGR or PGR (progesterone receptor) gene promoters become hypermethylated (30). The effects of histone acetylation or methylation at steroidogenic gene loci upon transcription depend on both the specific modification and the reproductive stage. The hypothalamic-pituitary axis experiences a negative feedback loop control by progesterone which regulates LH and FSH secretion patterns during the luteal phase for hormonal balance (125). The PGR-dependent gene networks mediate these processes while managing immune tolerance and vascularization within the CL which are necessary for pregnancy success. The secretion of gonadotropins receives additional refinement from estrogens working through ERα and ERβ receptors which affects both CL duration and functionality (126, 127). Mutations that affect hormone receptor genes together with epigenetic regulatory element methylation problems disrupt the highly complex endocrine dialog (29). The disturbances in hormone production cause luteal phase deficiency which results in inadequate progesterone levels that could lead to implantation failure and early pregnancy termination (128). The dynamic lifecycle of the corpus luteum integrates angiogenic, steroidogenic, and apoptotic processes that collectively determine its functional fate (**Figure 9**).

Although the AMPK-PPARγ-EZH2 axis has become a potentially relevant integrative system between metabolic sensing and epigenetic regulation in the corpus luteum (CL), existing evidence is context-dependent and in other cases indirect. The central cellular energy sensor, AMP-activated protein kinase (AMPK) has been demonstrated to have both synergistic and antagonistic impacts on luteal steroidogenesis. In response to metabolic or energetic stress, AMPK activity inhibits anabolic pathways, including cholesterol transport and progesterone biosynthesis, in part by regulation of steroidogenic proteins, including steroidogenic acute regulatory protein (STAR) and HSD3B (10). On the other hand, within certain physiological settings, AMPK can also be involved in the signaling pathway, including mTOR or peroxisome proliferator-activated receptor gamma (PPARγ) to enhance cell survival, lipid metabolism, and adaptive responses in luteal cells (**Table 4**). The role of PPARγ is pleiotropic and cell-type-specific, i.e. the control of lipid homeostasis and steroidogenic potential in ovarian cells, but its specific role in luteal activity differs between species and among experimental models (130, 131). Additionally, recent *in vitro* studies have shown that PPARβ/δ ligands regulate oxidative status and inflammatory responses in the inflamed corpus luteum, further supporting the role of PPAR signaling in luteal physiology (132). Proteomic studies have demonstrated that PPARγ ligands influence luteal protein networks associated with steroidogenesis and cellular metabolism, emphasizing the importance of metabolic regulation in corpus luteum function (133). Simultaneously, the catalytic subunit of the polycomb repressive complex 2 (PRC2), enhancer of zeste homolog 2 (EZH2) facilitates trimethylation of histone lysine 27 (H3K27me3), which is mainly linked with transcriptional suppression of steroidogenic and survival-related genes during luteal regression (134). Notably, the stage-specific and species-specific functional interplay of the AMPK, PPAR, and EZH2 in CL is not completely indicated. Consequently, additional studies with well-characterized *in vivo* and *in vitro* models are necessary to identify direct mechanistic evidence in comparison with extrapolative evidence and define the exact mechanism of control by this axis in luteal physiology. Importantly, these hormonal, metabolic, and epigenetic mechanisms do not function independently but operate in a highly interconnected manner to regulate luteal physiology.

**Table 4:** The context-dependent and species-specific functions of the PPARγ (gamma) regulatory axis in the corpus luteum activity.

Component	Species/model	Cell type	Effect/mechanism	Functional outcome	References
AMPK	Bovine CL	Luteal cells	Activation under energy stress inhibits STAR, HSD3B	↓ Progesterone synthesis	(135).
AMPK	Rodent ovary	Granulosa/luteal cells	Context-dependent interaction with mTOR signaling	Cell survival/metabolic adaptation	(136).
PPARγ	Human/rodent ovary	Granulosa/luteal cells	Regulates lipid metabolism and steroidogenic gene expression	Modulates steroidogenesis	(137).
EZH2	Mammalian CL	Luteal cells	H3K27me3-mediated chromatin repression	Gene silencing during luteolysis	(137).

### 4.2 Environmental and physiological stressors

The corpus luteum stands as a critical reproductive organ in female physiology because it demonstrates high sensitivity to environmental and physiological stress factors (129). External stressors damage both structural aspects and operational capacity of the corpus luteum through modifications of genetic and epigenetic regulatory systems (138). Heat stress together with nutritional imbalances serve as major factors influencing CL development duration and lifespan which affects both fertility and pregnancy stability (129, 139).

The worldwide temperature increase has turned environmental heat stress into a major issue that threatens livestock and human populations (140–142). The function of luteal tissue becomes compromised when ambient temperatures rise because they enhance reactive oxygen species (ROS) production in luteal cells. Oxidative stress damages cells while it reduces expression levels of essential steroidogenic genes HSD3B and STAR that are vital for progesterone synthesis (138). The accumulation of ROS due to heat exposure activates caspases which leads to luteal cell apoptosis and premature luteal cell death and luteolysis (143).

Heat stress modifies gene expression by inducing DNA hypomethylation and histone deacetylation events on the epigenetic level (144). Heat stress produces modifications that limit access to chromatin and block the transcription of genes needed for CL maintenance. The epigenetic silence of antioxidant response elements (AREs) allows the CL to become more vulnerable to ROS damage. Early CL regression occurs within an environment that reduces progesterone production and endangers early pregnancy maintenance (144, 145).

The epigenetic control of CL function depends significantly on physiological elements such as nutritional factors. The human body requires sufficient consumption of methyl-donating nutrients including folate and methionine and vitamin B12 to preserve normal DNA and histone methylation patterns (146, 147). Deficiencies of methyl-donating nutrients prevent enzymatic processes from maintaining proper methylation regulation which leads to hypomethylation of steroidogenesis-related genes STAR and PGR and angiogenesis-related gene VEGF (146). The condition leads to luteal insufficiency which results in poor vascular development together with insufficient hormone production. The CL receives epigenetic influence from micronutrients including zinc and selenium because they modulate both antioxidant enzymes and inflammatory mediators (148). Selenium increases expression of glutathione peroxidase antioxidant enzyme which protects luteal cells from ROS-induced apoptosis. Zinc controls immune system functions while its connection to metallothioneins has been established to protect against oxidative stress (149).

Research studies now demonstrate how microRNAs (miRNAs) function as small non-coding RNAs to control gene expression post-transcriptionally in CL physiology (150, 151). The expression levels of miRNAs respond to nutritional elements that subsequently control gene translation of important luteal genes that produce steroids and support cell survival and vascular development (106). Nutritional treatments showing potential to enhance reproductive health can achieve success through the specific modulation of epigenetic processes.

### 4.3 Immune system and inflammation

The corpus luteum functions as both an endocrine and immunological structure which experiences substantial morphological changes during its existence (152). The ovulated follicle attracts multiple immune cells such as macrophages and dendritic cells and T lymphocytes that support the maintenance of luteal function and structure. Immune components play essential roles in tissue remodeling and angiogenesis and luteal cell survival thus enabling progesterone production and the support of early pregnancy (153).

The immune cells release three important cytokines including tumor necrosis factor-alpha (TNF-α), interleukin-1 beta (IL-1β), and interferon-gamma (IFN-γ) which control luteal activity throughout different phases (154). These cytokines activate important processes of angiogenesis and extracellular matrix remodeling in the early luteal phase to establish a functional CL (155). The late luteal phase shows increased cytokine expression that leads to luteal cell apoptosis and vascular regression to facilitate luteolysis (155, 156). During the luteal cycle the expression of cytokines and their receptors undergoes detailed genetic transcriptional control which varies according to the cycle phase (157). The regulatory mechanisms protect inflammatory processes from becoming harmful to the system. Epigenetic mechanisms regulate both the immune mediators and their regulatory processes. The immune response in the CL environment receives precise control through histone modifications which include histone 3 lysine 4 trimethylation (H3K4me3) and histone 3 lysine 27 trimethylation (H3K27me3) (158) that either activate or repress cytokine gene expression (98).

Any damage to this refined immune equilibrium creates harmful reactions. The homeostatic balance of immunity becomes disrupted when infections or autoimmune conditions or epigenetic modifications of cytokine genes occur (159). This results in either over activating the immune response (160). The abnormal immune system equilibrium can cause CL tissue to regress prematurely and reduce progesterone production which may lead to pregnancy termination (161). When TNF-α is overexpressed beyond epigenetic control mechanisms it causes excessive cell death in luteal cells (162). Knowledge about how immune signaling cooperates with epigenetic regulation in the CL shows useful information about reproductive disorders. Medical experts should create targeted therapies that control luteal function for women experiencing recurrent pregnancy loss and luteal phase deficiency and immune-related fertility problems.

## 5 Implications of CL dysfunction on pregnancy outcomes

### 5.1 Impact on implantation and early pregnancy loss

The corpus luteum (CL) acts as a vital organ for early pregnancy development because it produces progesterone which prepares the endometrium for embryo implantation and sustains the pregnancy until placental functions become active (127). The endometrium undergoes decidualization under progesterone influence while the hormone regulates implantation-associated gene expression that includes HOXA10 (163) and leukemia inhibitory factor (LIF) (164) and different integrins which help create receptive endometrium conditions for blastocyst adhesion (165).

Insufficient progesterone production from CL dysfunction leads to the condition known as luteal phase deficiency (LPD) (166). Insufficient progesterone levels create a severe impairment that damages endometrial transformation capability thus making the endometrium unsuitable for embryo implantation (167). A developing embryo faces reduced support when the uterine lining fails to receive sufficient progesterone support because the endometrium either remains thin or shows poor vascular development. The exact timing of implantation becomes disturbed when these conditions occur which increases both implantation failure risks and embryonic losses early in pregnancy (168).

The maternal immune environment suffers adverse effects when progesterone levels become insufficient because of dysfunctional corpus luteum function (169). The immunomodulatory properties of progesterone help the mother accept the semi-allogenic embryo through maternal immune tolerance. The insufficient production of progesterone triggers increased activity of uterine natural killer cells and elevated cytokine levels and diminished regulatory T cell function which together endanger embryonic survival and increase the risk of recurrent early pregnancy loss (170). The scientific community now investigates extracellular vesicles (EVs) including exosomes from the CL (171) because these vesicles facilitate communication between the ovary and uterus (172). The bioactive molecules contained in vesicles include proteins and mRNAs as well as microRNAs that control endometrial receptivity and implantation readiness (173). These alterations in EV production by dysfunctional corpus luteum impair essential communication signals that lead to reduced implantation probability.

The multiple issues associated with CL dysfunction create serious harm to early pregnancy through its impact on endocrine function and immune regulation and organ-to-organ signaling capabilities (174). Early pregnancy loss together with implantation failure occurs due to these disruptions which demonstrate why reproductive health management needs early corpus luteum assessment (**Table 5**) (179).

**Table 5:** Impact of CL dysfunction on early pregnancy outcomes.

Condition	Hormonal/physiological impact	Clinical manifestations	Potential outcomes	References
Luteal Phase Deficiency (LPD)	Insufficient progesterone production from the CL	Short luteal phase, inadequate endometrial preparation for implantation	Increased risk of implantation failure and early pregnancy loss	(166)
Recurrent Pregnancy Loss (RPL)	Reduced CL vascularization, decreased expression of angiogenic factors like VEGF and ANGPT2	Multiple consecutive miscarriages	Higher incidence of early pregnancy loss	(175)
Preterm Birth & IUGR	Inadequate early progesterone levels, defective placental support, pro-inflammatory uterine conditions	Premature delivery, intrauterine growth restriction	Increased risk of adverse neonatal outcomes	(176)
Preeclampsia	Impaired trophoblastic invasion, epigenetic dysregulation of immune and vascular genes	Hypertension, proteinuria, organ dysfunction	Potential maternal and fetal morbidity	(177, 178)

### 5.2 Role of inadequate CL function in pregnancy disorders

The corpus luteum functions as the primary support system for maintaining early pregnancy thus inadequate CL function leads to multiple pregnancy complications (21). RPL stands as one of the best documented associations with recurrent pregnancy loss which describes the loss of two or more consecutive pregnancies. Medical research shows that luteal insufficiency stands as a main contributing cause in women who experience recurrent pregnancy loss (180).

RPL patients show reduced CL vascularization during histological and biochemical assessments because poor vascularization hinders the transport of cholesterol precursors and other substrates vital for progesterone production. The impaired luteal tissue function results from decreased expression of angiogenic factors especially vascular endothelial growth factor (VEGF) and angiopoietin-2 (ANGPT2) (181). Adequate vascular support is needed for luteal tissue to produce hormones properly which leads to poor endometrial receptivity and embryonic development.

DNA variations known as single nucleotide polymorphisms (SNPs) in CYP11A1 and HSD3B1 steroidogenesis-related genes make people more susceptible to CL functional problems (182). The genes perform essential functions in cholesterol-to-progesterone conversion while mutations create conditions that diminish hormone synthesis capability. Angiogenic pathway genes which undergo alterations lead to decreased formation of vascular support structures inside the CL (183).

Epigenetic processes work alongside other mechanisms to control the functional operations of the luteal tissue. The hypermethylation of progesterone receptor (PGR) gene in luteal cells leads to receptor expression loss which impairs hormone response and breaks down the regulatory feedback needed for sustained hormone production (184). The steroidogenesis process and luteal cell differentiation suffer from adverse effects due to abnormal microRNA expression patterns of miR-132 and miR-200c thus worsening hormonal insufficiency (185). Inadequate CL function now stands recognized as a leading cause of serious pregnancy complications which also include preterm birth together with intrauterine growth restriction (IUGR) (186). Early pregnancy progesterone levels that are insufficient to properly develop the uterine lining alongside inadequate placental support occur in such cases. Pro-inflammatory conditions develop inside the uterus while vascular remodeling becomes defective due to these processes which are necessary for placental invasion and fetal nourishment (187).

Research shows that inadequate early progesterone levels lead to shallow trophoblastic invasion which occurs similarly in preeclampsia cases (188). The conditions produce epigenetic dysregulation that affects genes which control immune modulation and vascular growth and trophoblast function. The immune response to fetal development could become unfavorable when IL-10 and TGF-β gene promoters experience hypomethylation or histone modifications (189). At the same time VEGF-related gene regulation changes lead to reduced uteroplacental blood flow (190).

### 5.3 Genetic and epigenetic biomarkers for CL-related pregnancy complications

The research for dependable biomarkers to evaluate corpus luteum function intensifies because this structure plays an essential role during early pregnancy. The medical community recognizes genetic and epigenetic markers as potential diagnostic tools for luteal insufficiency with predictive capabilities toward recurrent pregnancy loss (RPL) and preeclampsia alongside intrauterine growth restriction (IUGR) (191). Such biomarkers promise to develop precision medicine techniques and help medical staff identify high-risk pregnancies before they progress and therefore make interventions quicker and more effective (192).

Geneticists study polymorphisms of genes that control progesterone synthesis alongside genes that control angiogenesis development for their potential predictive power. A STAR (Steroidogenic Acute Regulatory Protein) gene variation causes problems with cholesterol transport in luteal cells which decreases progesterone production (193). The balance between estrogen and progesterone as well as hormonal sensitivity depends on PGR (progesterone receptor) and CYP19A1 (aromatase) polymorphisms which affect optimal CL function (194). The clinically relevant variants discovered among genes that control angiogenesis include VEGFA (vascular endothelial growth factor A) and FLT1 (VEGFR1) (195). The genetic variations create problems for luteal vascularization leading to poor delivery of production substrates that negatively affect endometrial receptivity. Different from static genetic variants provide epigenetic biomarkers to monitor changes in gene expression which happen because of physiological and environmental stress. The DNA methylation patterns in PGR, FOXO1 (Forkhead Box O1) and inflammatory cytokines including IL-1β, TNF-α impact CL dysfunction leading to adverse pregnancy outcomes (196). Promoter hypermethylation of PGR reduces receptor expression which hinders the CL from sustaining progesterone production. A change in FOXO1 transcription factor promoter methylation could affect both decidualization of the endometrium and the correct functioning of luteal cells during early pregnancy (38).

The CL experiences gene expression changes through histone modification processes that include acetylation and methylation changes in histone tails. Luteolysis and pregnancy failure develop when key steroidogenic and angiogenic genes become silenced due to abnormal profiles of H3K9 acetylation decrease and H3K27 trimethylation increase (197). The epigenetic modifications provide therapeutic opportunities to enhance CL function by using drugs which modify histone patterns or administer dietary substances that change epigenetic expressions (198).

Modern research has demonstrated the potential value of circulating microRNAs (miRNAs) as non-invasive biomarkers which track CL health status. Luteal cells emit miR-21, miR-210 and miR-155 miRNAs into bloodstreams and extracellular vesicles (199). CL function and luteal stress systemic response into blood circulation cause changes in these microRNA expression levels. The hypoxia-inducible miRNA miR-210 demonstrates elevated levels during poor vascularization of the corpus luteum which leads to inhibition of angiogenesis-related gene networks (200).

The clinical screening protocols should include these validated genetic and epigenetic biomarkers to detect luteal insufficiency in early stages (201). The biomarkers create opportunities for therapists to develop targeted therapeutic interventions like progesterone supplementation and angiogenic therapy together with epigenetic modulation that match the molecular reasons for dysfunctional CL (202). Biomarker-based management systems present a strong opportunity to enhance pregnancy results for women who experience luteal phase abnormalities.

## 6 Potential therapeutic strategies for CL dysfunction

### 6.1 Hormonal interventions

The successful management of corpus luteum (CL) insufficiency requires hormonal treatments as the main strategy to enable implantation and sustain early pregnancy (21). Exogenous administration of progesterone stands as the primary treatment method to substitute insufficient endogenous hormone production. The intervention becomes vital during the luteal phase and early gestation because constant progesterone levels are crucial for endometrium transformation into a receptive state and maintaining early embryonic development (203).

Doctors use different clinical protocols for progesterone treatment which they personalize for each patient undergoing fertility treatments. The medical community prefers vaginal micronized progesterone because it delivers localized treatment while producing minimal side effects in the rest of the body (204). Medical practitioners use intramuscular progesterone injections because they provide stable serum levels which makes them suitable for *in vitro* fertilization (IVF) cycles (205). The absorption of oral progesterone medications becomes inconsistent because of first-pass metabolism which leads to reduced effectiveness. The administration of progesterone through any route performs three essential functions by maintaining endometrial receptiveness and controlling myometrial contractions and regulating maternal immune responses through cytokine regulation and immune cell activity modulation (206, 207).

The hormonal approach uses multiple methods to strengthen the body’s progesterone production system. Human chorionic gonadotropin (hCG) serves as a common treatment in ART protocols after ovulation or oocyte retrieval (208). The hormone hCG functions like luteinizing hormone (LH) to activate the corpus luteum production of progesterone and extend its lifespan. The treatment risks ovarian hyperstimulation syndrome (OHSS) mainly affects patients with high ovarian responses (209). Gonadotropin-releasing hormone (GnRH) agonists serve as an alternative method because they cause endogenous LH surges to maintain CL function (210). The medical protocol uses these medications in changed natural and antagonist ART cycles which have reduced LH support. These agents help create the needed hormonal environment for normal luteal functioning especially when CL disruption occurs because of medical procedures (211).

### 6.2 Epigenetic therapies

Epigenetic treatments offer an alternative method to treat dysfunction of corpus luteum (CL) by altering gene expression patterns without altering the DNA sequence (212). Recent scientific evidence indicates that in luteal cells, abnormal DNA methylation and histone changes lead to significant issues with the expression of key genes involved in progesterone production and genes required to perform angiogenesis and cell survival (213, 214). Medical research focuses on the possible therapeutic effects of epigenetic modification targeting agents in the treatment of reproductive disorders related to CL dysfunction. The DNA methyltransferase inhibitor group (DNMTi) offers potential epigenetic agents with 5-azacytidine and decitabine as its representatives (215). The cytidine analogs penetrate the DNA structure and inhibit the action of DNA methyltransferase leading to passive and active promoter demethylation.

The administration of DNMTi agents restores the function of essential genes STAR (steroidogenic acute regulatory protein) and PGR (progesterone receptor) and VEGFA (vascular endothelial growth factor A) which leads to the recovery of luteal steroidogenesis and vascular integrity in cases of CL dysfunction (216). Research on reproductive tissues through preclinical models has begun to demonstrate the potential of these agents to treat epigenetically mediated luteal insufficiency even though their primary use remains cancer treatment (217).

The histone deacetylase inhibitor drugs trichostatin A, vorinostat, and valproic acid demonstrate great potential among promising therapeutic categories (218). HDAC inhibitors work through boosting histone acetylation to make chromatin more flexible and allow target genes to become transcribed. Experimental studies demonstrate that HDACi increase pro-angiogenic factor ANGPT1 and VEGF and steroidogenic enzyme CYP11A1 and HSD3B1 expression in the CL leading to better luteal vascularization and hormone production (219, 220). The agents modify the immune microenvironment of the CL by controlling the expression patterns of cytokines and immune modulators that sustain a supportive cellular environment for luteal cells.

Therapeutic research is now being conducted regarding epigenetic regulation of non-coding RNAs with a focus on microRNAs (miRNAs). Scientific research shows that the abnormal development of the luteal tissue along with impaired progesterone production relates to dysregulated expression of miR-132, miR-210, and miR-155 (221). The targeted therapy of CL dysfunction through post-transcriptional adjustments can be achieved by altering miRNA expression using mimics or antagomirs.

The potential of these therapeutic approaches encounters multiple ongoing difficulties. The administration of epigenetic drugs leads to unintended effects which modify gene expression throughout different body tissues (222). Due to the changing epigenetic control during the reproductive cycle the right implementation timings and dosages of these treatments become essential. The present use of these therapies remains restricted in human reproductive medicine because of both ethical and safety factors (223). The expanding knowledge about CL epigenetic landscapes will enable these therapies to serve as essential components for personalized medical treatments of luteal insufficiency and other pregnancy disorders. Researchers must conduct more in-depth investigations including direct studies on living organisms and clinical testing to create secure and efficient reproductive healthcare methods from experimental outcomes. Additionally, the lack of tissue-specific targeting and the risk of long-term epigenetic alterations remain significant challenges for their safe clinical translation.

### 6.3 Nutritional and lifestyle interventions

Improving corpus luteum function and reducing the reproductive issues become more common in clinical practice with the adoption of nutritional and lifestyle methods. The interventions improve fertility because they maintain early pregnancy by supporting the luteal microenvironment through antioxidant benefits and hormonal balance functions and epigenetic adaptations (224). Resveratrol stands out as a naturally occurring polyphenolic compound which exists in grapes berries and red wine for its beneficial effects on luteal health (225). The compound activates metabolic and epigenetic regulators SIRT1 and PGC-1α which lead to mitochondrial biogenesis and reduce oxidative stress and promote vascular development (226) as part of their constitutive actions.

Resveratrol is a compound that leads to angiogenesis and progesterone production to maintain the adequate functioning of the CL. This chemical regulates the expression patterns of genes by acetylating histones and, at the same time, regulates the molecules by DNA methylation (225). Protective effects of other polyphenols, such as curcumin (turmeric) and quercetin (apples, onions, capers), on ovarian and luteal tissues also occur. The compounds have been reported to inhibit pro-inflammatory cytokines, alleviate oxidative stress and affect epigenetic modifications including histone acetylation and microRNA expression. The combined effects of them lead to improved luteal vascularization and steroidogenic production (227).

It is a well-documented cause of CL dysfunction, which is oxidative stress, or the excessive generation of reactive oxygen species (ROS) (228). ROS disrupt the integrity of the mitochondrion, inhibit steroidogenic enzymes (CYP11A1 and HSD3B1) and initiate apoptosis of luteal cells (229). These effects can be countered by antioxidant supplementation. Vitamin C and E eliminate ROS and enhance the resilience of cells. Coenzyme Q10 is a mitochondrial co-factor that helps in energy production and repair processes in the cells (230). N-acetylcysteine (NAC) enhances glutathione synthesis, providing critical intracellular antioxidant defense. These agents collectively promote luteal cell survival and sustained progesterone secretion (231).

Change in lifestyle has a significant impact on hypothalamic-pituitary-ovarian (HPO) axis and thus controls ovarian cyclicity and corpora lutea (CL) activity in mammals. Prolonged stress significantly increases cortisol levels, which also have the potential to inhibit gonadotropin-releasing hormone (GnRH) and luteinizing hormone (LH) pulses (232), thus disrupting the formation and upkeep of CL. Mindfulness meditation, yoga and physical exercise are all types of interventions that have been shown to reduce stress biomarkers, boost mood and restore endocrine homeostasis. In addition, proper sleep is essential to the normalization of hormones; sleep disorders are linked to irregular periods and luteal deficiency (233). Eating habits also influence reproduction. Anti-inflammatory diets containing omega-3 fatty acids, whole grains, leafy vegetables, and lean proteins are associated with anti-inflammatory pathways and provide the required micronutrients (zinc, selenium, and folate) essential to DNA methylation and luteal hormone production (234). On the other hand, excess consumption of transfats, refined sugars, and processed foods worsen the situation of inflammation and hormonal imbalance.

## 7 Conclusion

The corpus luteum (CL) is a temporary but essential endocrine gland, the coordinated development, activity, and recession of which is the key to reproductive success due to prolonged production of progesterone. This review explores existing evidence that shows that CL biology is regulated by a series of tightly integrated genetic, hormonal, metabolic, and epigenetic regulatory networks in mammalian species. The molecular basis of lutealization and progesterone synthesis is the presence of core steroidogenic genes (STAR, CYP11A1, LHCGR, and PGR) and transcriptional regulators (SF-1, FOXO1 and LRH-1), whereas endocrine signaling (MAPK/ERK, PI3K/AKT, and WNT/β-catenin) fine-tune luteal cell survival, angiogenesis, In addition to classical hormonal regulation, new evidence also indicates that metabolic-epigenetic integration is central in the process of defining luteal fate, where AMPK can serve as an important sensor of energy, PPARγ can be a context-specific transcriptional regulator of lipid metabolism and cell fate choices, and H3K27me3, which is mediated by EZH2, is a stable manner in which steroidogenic genes are repressed in luteal regression. The disruption of these interconnected pathways is involved in luteal insufficiency, impaired progesterone synthesis and poor reproductive success, such as implantation loss and premature pregnancy loss, in both humans and livestock species valuable for economic purposes. The further insight of the cooperative relations between genetic, metabolism, and epigenetic controllers, and especially the AMPK-PPARγ-EZH2 axis, provides a conceptual base of further biomarker discovery and targeted therapy, with the potential to use it in the enhanced fertility regulation and reproductive performance in clinical and farming practices.
